# Psoriasis and Respiratory Comorbidities: The Added Value of Fraction of Exhaled Nitric Oxide as a New Method to Detect, Evaluate, and Monitor Psoriatic Systemic Involvement and Therapeutic Efficacy

**DOI:** 10.1155/2018/3140682

**Published:** 2018-09-23

**Authors:** Pierachille Santus, Maurizio Rizzi, Dejan Radovanovic, Andrea Airoldi, Andrea Cristiano, Rosalynn Conic, Stephen Petrou, Paolo Daniele Maria Pigatto, Nicola Bragazzi, Delia Colombo, Mohamad Goldust, Giovanni Damiani

**Affiliations:** ^1^Department of Biomedical Sciences L. Sacco, University of Milan, Milan, Italy; ^2^Respiratory Unit, Center for Sleep and Respiratory Disorders “Luigi Sacco” University Hospital, Milan, Italy; ^3^Department of Dermatology, Case Western Reserve University, Cleveland, OH, USA; ^4^Department of Emergency Medicine, Good Samaritan Hospital Medical Center, New York, USA; ^5^Clinical Dermatology, Department of Biomedical, Surgical and Dental Sciences, IRCCS Galeazzi Orthopaedic Institute, University of Milan, 20126 Milan, Italy; ^6^Department of Health Sciences (DISSAL), School of Public Health, University of Genoa, 16132 Genoa, Italy; ^7^Department of Pharmacology, University of Milan, Milan, Italy; ^8^Mazandaran University of Medical Sciences, Sari, Iran; ^9^Dipartimento di Fisiopatologia Medico-Chirurgica e dei Trapianti, Università degli Studi di Milano, Unità Operativa di Dermatologia, IRCCS Fondazione Ca' Granda, Ospedale Maggiore Policlinico, Milano, Italy; ^10^Young Dermatologists Italian Network (YDIN), GISED, Bergamo, Italy

## Abstract

Psoriasis is a chronic inflammatory systemic disease characterized by a wide range of comorbidities. Respiratory comorbidities are currently poorly characterized and with discordant results. The systemic state of inflammation caused by psoriasis acts de novo on respiratory tissues and amplifies preexisting inflammation from asthma or chronic obstructive pulmonary disease. Because the lungs act as a gas exchanger between the internal and external environment, the impact of chronic psoriasis inflammation may be easily assessed through the analysis of exhaled breath. The fraction of exhaled nitric oxide test (FeNO) is a potential noninvasive solution that can provide quantitative and qualitative indices of respiratory airway inflammation. FeNO is routinely used to screen and manage asthmatic patients. Recent pilot studies contain encouraging data that underscore its possible use with systemic inflammatory nonpulmonary diseases, such as psoriasis. FeNO may therefore be a useful tool to evaluate underestimated airway inflammation and at the same time globally evaluate the impact of systemically antipsoriatic therapies.

## 1. Introduction

Psoriasis (PS) is a chronic inflammatory systemic disease [1–4], characterized by scaling and erythematous plaques. It affects 2-3% of the present population [5]. From its relapsing remitting behaviour to related comorbidities, many bodies of evidence have proposed the idea that psoriasis extends beyond the skin [6]. Literature has shown PS-systemic inflammation is associated with higher risk of developing depression [7], cardiovascular events [8], diabetes [9], and obstructive sleep apnea syndrome (OSAS) [10, 11]. Respiratory comorbidities are currently poorly characterized with conflicting findings [12–14]. Difficulties in assessing lung comorbidities in PS patients may be categorized into two main groups: (1) low compliance of PS patients to be extensively assessed [15]; (2) lack of a validated noninvasive, inexpensive, convenient tool. Educating patients that psoriasis not only affects the skin but also targets joints [16], entheses [17, 18], and the entire body by maintaining a chronic inflammatory status can help resolve problems with patient compliance. Creating a noninvasive assessment tool for potential lung comorbidities is the main focus of this review. Multiple epidemiological studies outline a possible correlation between PS and respiratory diseases [12–14]. They highlight an increased risk of asthma, chronic obstructive pulmonary disease (COPD), and airway infections in PS patients [14]. These diseases share common inflammatory pathogenic pathways with other psoriasis comorbidities [8]. Although the exact mechanism of PS comorbidities is still unclear, the most accepted hypothesis is that proinflammatory cytokines spill over through the blood from active psoriatic plaques, causing inflammation in distant tissues [19–21]. Each tissue responds in a specific way to the insult, such as atherosclerosis in the coronary and carotid vessel [22]. In addition, genetically predisposed individuals [23], whose proinflammatory cytokines trigger long distance inflammation, may exhibit consolidation and auto-maintenance [24]. This pathogenic theory designated as the “psoriatic march” was postulated by Boehncke. It attempts to explain the epidemiological correlation of psoriasis and cardiovascular comorbidities linked by a mechanism driven by systemic inflammation [24]. This idea could theoretically be applicable to all PS comorbidities, including respiratory. In fact, recent literature has described impaired lung function in psoriatic patients exhibiting lower mean Force Expiratory Volume in the 1^st^ second (FEV1)/Force Vital Capacity (FVC) ratios and Forced Expiratory Flow (FEF) 25-75% [25]. Biological data confirms this theory, stating there is an increased tissue sensitivity to several proinflammatory cytokines including tumour necrosis factor-*α* (TNF*α*) and interleukin-1 (IL-1)[26]. This has been observed in psoriasis plaques and the blood of PS patients [26]. These cytokines act locally, in an autocrine or paracrine way, to maintain endocrine-like behaviour. If present in high enough concentration cytokines may also manifest endocrine-like behaviour, causing inflammation in other body areas [27]. This includes the airways through nuclear factor kappa-light-chain-enhancer of activated B cells (NF*κ*B) signaling transcription of several key enzymes, such as the inducible form of nitric oxide synthase (iNOS) which produces nitric oxide (NO) [28]. Because the lungs act as a gas exchanger between the internal and external environment, the impact of chronic psoriasis inflammation may be assessed by the analysis of fraction of exhaled nitric oxide (FeNO). FeNO could provide a quantitative and qualitative indicator of respiratory airway inflammation [29], as it is routinely used to screen and manage asthmatic patients [30]. Pilot studies have highlighted the possible use of FeNO in systemic inflammatory nonpulmonary diseases [Table 1] [31–34], such as psoriasis. In this review, we describe the rationale and possible applications of FeNO in the management of PS.

## 2. Asthma and Psoriasis

Previous studies suggested a possible link between asthma and psoriasis [12–14]. Furthermore, asthma may be considered an umbrella term that groups different patterns of airway inflammation: paucigranulocytic (≤3% eosinophils, <64% neutrophils) and eosinophilic (>3% eosinophils, <64% neutrophils) without increased total cell counts, neutrophilic (≤3% eosinophils, ≥64% neutrophils) and mixed granulocytic (>3% eosinophils, ≥64% neutrophils) [63]. Although Th2 cells have a well-defined role in eosinophilic inflammation initiated by releasing IL-4, IL-5, and IL-13 and Th1 and Th17 cells may also lead to the neutrophilic response by releasing IL-6, IL-12, IL-17, and IL-23[64]. IL-23 itself has been suggested to mediate neutrophilic antigen-mediated recruitment and at the same time eosinophilic recruitment via Th2 in the airways [65]. The pathophysiology is also enriched by several overlapping genetic risk loci that link psoriasis to asthma [66–68]. A recent meta-analysis described that asthma risk in PS patients is higher and that patients >50 years old have the highest risk [69], due to the increased duration of long-term inflammation in the airways.

Nadeem et al. have recreated in a mouse model the coexistence of psoriasis and asthma by triggering psoriasis with topical application of imiquimod and asthma by sensitizing mice airways with cockroach extract [70]. Mice treated in this manner had an increased count of both neutrophils and eosinophils in the airways, suggesting that psoriasis may increase airway inflammatory pattern [70]. This hypothesis was ultimately confirmed in these mice by the increased number of both Th17 and Th2-specific cells as identified by STAT3-RORC and GATA3 [70]. Interestingly a topical inhibitor of STAT3 decreased both Th17 and Th2 counts, suggesting that Th17 have a prominent role in triggering and modulating inflammation in both the skin and the lung [70]. A unique case reports IL-12∖IL-23 inhibitors as effective in treating a patient affected by both psoriasis and asthma [71].

## 3. COPD and Psoriasis

The relationship between COPD and psoriasis is highlighted by two distinct meta-analyses that suggest an association between these two chronic inflammatory diseases [72, 73]. In particular Ungprasert et al. found that COPD in PS patients has an odds ratio of 1.45 (95% CI, 1,21-1,73) by evaluating a total of 7 studies and 331,347 patients [74–80]. Recently Wu and colleagues analysed 110,729 incidental cases of psoriasis and their comorbidities, clustering the entire sample in 4 classes; one which manifests a propensity to develop COPD and hypertension [81]. Despite the nature of immune system dysfunction in COPD remaining largely unknown, the crosstalk between innate and adaptive immunity has been shown previously to lead to increased severity [82]. Dysfunctional neutrophils chemoattraction lead to a premature diapedesis and neutrophilic elastase release that inhibits dendritic cells (DCs) maturation, increased mucin production, and decreased lung function [83]. The active respiratory epithelium releases MIP3*α*/CCL20 that informs and recruits Th17 and DCs [84]. In fact, in COPD, as well as in psoriasis, patients demonstrate an imbalance between Th17 and Treg, a finding confirmed by Wang and colleagues [84]. High levels of Th17 cells in small airway associate with poor function; in particular, IL-17A and IL-17F trigger and lead to development of neutrophilic airway inflammation [83]. This inflammatory pattern was also tested by Nadeem and colleagues who found that psoriasis triggers and is triggered by airway inflammation [70].

## 4. Exhaled Nitric Oxide (FeNO) Measurement

All NO synthase (NOS) isoenzymes convert L-arginine to L-citrulline with the generation of NO [85]. Constitutive NOS includes neuronal-NOS (NOS1 or nNOS), endothelial-NOS (NOS3 or eNOS), and mitochondrial-NOS (mtNOS) [86, 87]. Inducible-NOS (iNOS or NOS2) is expressed only in response to inflammatory and infectious triggers, producing large quantities of NO independently by calcium ion influx [88]. NO in the lungs is mainly produced by the alveoli epithelium and moves to the lumen driven by a concentration gradient. Consequently, FeNO is a flow-dependent measure [89]. The gold standard to assess FeNO is an inline method by which expiration is continuously sampled by an NO analyser. The resultant NO profile versus time or exhaled volume together with other exhalation variables (airway flow rate and/or pressure) is captured and displayed in real-time [89]. The patient inhales NO sampled air through a preassessed calibrated mouthpiece up to total lung capacity and then exhales without holding their breath, as a breath hold would increase NO diffusion time from the airway wall to air and cause inaccurate results. It is for this same reason that low exhalation flow rates increase FeNO and high ones decrease them. It is common practice to display the pressure or expiratory flow rate to the subject, who is subsequently asked to maintain a positive mouthpiece pressure between 5 and 20 cmH2O to avoid nasal backflow of NO. A nose clip should also be avoided. Correct exhalation should last for >6 seconds (exhaled volume of at least 0.3 L at 50 mL/s) resulting in a single-breath NO profile (exhaled NO versus time plot) that consists of a washout phase followed by a plateau of at least 3 seconds. Repeated exhalations should be performed to obtain at least 2 values that agree within 10% of each other. Exhaled NO is then calculated as the mean of the two values. At least 30 seconds of tidal breathing off the circuit should elapse between exhalations [89]. FeNO obtained at 50 mL/s is a reliable source of the production of NO in respiratory airways, but there is no simple surrogate for determining the distal airway/alveolar concentration of NO (CANO), because FeNO must be obtained at multiple expiratory flow rates in order to be able to distinguish between the airway production and CANO: 30, 50, 100, and 200 mL/s [89]. Physiological and pathological conditions responsible for affecting FeNO measurements are reviewed and summarized in Table 2 [35–62]. According to the American Thoracic Society (ATS)/European Respiratory Society (ERS) guidelines, nondisease related patient factors influencing FeNO values are very young age (children), female sex (during menses or pregnancy), respiratory maneuvers (performing spirometry reduces FeNO), airway calibrating, some food and beverages, smoking (which reduces FeNO chronically and acutely), infections, hypoxia, exercise, and medications directly related to NO synthesis [89] (Table 2).

## 5. Nitric Oxide as a Mediator of Inflammatory Disease

Initial pulmonary changes are not always evident from a classic spirometry exam. In these conditions, FeNO provides an added value in the early diagnosis of an active inflammatory process [90]. FeNO has already been accepted in literature as an indirect marker of airway inflammation used to monitor/manage asthma and prevent its recurrences [91]. Measurement of FeNO has also been suggested as a biomarker of several nonpulmonary diseases (Table 1). Different theories have been suggested to explain why FeNO measurement should be increased in nonpulmonary diseases: (1) leukocyte priming and subsequent homing in embryonically related tissues (as between lung and bowel) [92, 93] and (2) proinflammatory cytokine release from lesioned tissue driven by blood to the lung [19]. An increasing body of evidence has claimed the clinical usefulness of this method in daily practice as well as in nonrespiratory diseases. Decreased FeNO in heart disease may suggest a failure in compensatory circulatory mechanisms, as found in congestive heart failure (CHF) [93] and pulmonary hypertension (PH). Low alveolar NO is considered a marker of hypoxia and endothelial dysfunction since hypoxia induces vasoconstriction [85]. The same explanatory mechanism is also described in obstructive sleep apnea syndrome (OSAS) where the alveolar component (CANO) is decreased [94], most likely subsequent to hypoxia [95]. Although CANO is decreased in OSAS, overall FeNO is increased because the main component is produced by the upper airways [96]. Conversely, increased FeNO levels might reflect excessive vasodilation as in hepatopulmonary syndrome (HPS) [97] or be the consequence of a systemic high level of inflammatory cytokines as in Hodgkin's Lymphoma (HL) [98], systemic sclerosis (SSc) [99], Crohn's disease (CD) [91], and other inflammatory bowel diseases (IBDs) [92]. Perturbation of metabolism, such as in obesity, that leads to a proinflammatory condition may elevate FeNO as demonstrated by the correlation between body mass index (BMI) and FeNO [47].

## 6. Psoriasis and Nitric Oxide Production

The skin of psoriatic patients, both lesional and nonlesional, globally presents increased proinflammatory cytokine patterns, characterized by the presence of IFN*γ*, TNF*α*, IL-8, IL-1 and IL-6, and IL-17 [100–102]. NO is a labile mediator and can be detected at high levels in psoriatic plaques in the presence of these cytokines which results in overtranscription of iNOS and arginase through NF*κ*B signaling [103]. Although at low concentrations NO is physiologically and continuously released by the respiratory epithelium and endothelium [28], to enable high concentrations of NO release a stimulus is needed [104, 105]. Psoriasis causes endothelial vessel dysfunction [104] and an accumulation of asymmetrical dimethyl arginine (ADMA), NOS inhibitors [106], and decreased free NO content in blood [107]. At the same time, blood transports the locally produced proinflammatory cytokines that trigger inflammation in distant tissues. The production of NO is increased in many skin diseases such as contact dermatitis, atopic dermatitis, and psoriasis [108–111]. The increased levels of NO suggest a role for this molecule in maintaining the proinflammatory microenvironment of psoriasis. NO is described as capable of initiating psoriasis mainly by releasing calcitonin gene-related peptide and substance P. These substances are considered to play important roles in the pathogenesis of psoriasis by inducing the production of adhesion molecules, keratinocyte hyperproliferation, mast cell degranulation, vasodilatation, and chemotaxis of neutrophils [112]. NO stimulates epithelial cells to release chemokines and growth factors which appear to be important for keratinocyte proliferation and angiogenesis [113]. In psoriatic lesions, an overexpression of iNOS is associated with a compensatory increase of arginase-1 enzyme which may reduce the NO availability [114–120]. On the other hand, direct measurements of NO production in psoriatic lesions did not show any evidence of competitive inhibition [117]. In psoriasis patients, serum NO levels correlated with the Psoriasis Area Severity Index (PASI) [121] highlighting an important relationship between active disease and an increased level of FeNO [19]. Recently, a positive correlation between the PASI score and FeNO values has also been found at a flow of 50 mL s^−1^, which would reveal the presence of subclinical respiratory inflammation in patients with psoriasis [19]. Persistent inflammation may produce a change in airway microstructure, decreasing dynamicity and functionality [122]. Microstructure change may reflect a loss of function and a risk of developing respiratory disease. A demonstration of chronic subclinical inflammation could be useful in identifying the underlying biological mechanism contributing to the aetiology of chronically compromised respiratory function such as COPD [122] or OSAS [10]. It is noteworthy that psoriasis, COPD, and OSAS present some common risk factors such as obesity, smoking, physical inactivity, and metabolic syndrome. The vast majority of current studies are not prospective which prevents a clear establishment of a causal relationship.

## 7. Conclusion

Measurement of FeNO is an easy to perform, noninvasive, reproducible test which requires relatively low collaboration. The current available data seem to suggest that the proinflammatory systemic status due to psoriasis [123] may involve also the respiratory tract [124]. From this perspective, FeNO should be a part of screening moderate-to-severe psoriasis patients. The studies published to date encourage its introduction in daily practice and its inclusion into research of new biomarkers [19, 125]. Further studies are needed to validate existing data on FeNO measurement and to firmly establish it as part of standard screening in patients with moderate-to-severe psoriasis.

## Figures and Tables

**Table 1:** Fractions of exhaled nitric oxide display increased levels in autoimmune and inflammatory systemic disease.

**FRACTION OF EXHALED NITRIC OXIDE (FENO) IN AUTOIMMUNE/INFLAMMATORY DISEASES**		
**DISEASE**	**FENO**	**STUDIES**
**Psoriasis**	↑	Malerba M, et al. [19]
		Damiani G, et al. [20]
		Malerba M, et al. [21]
**Juvenile idiopathic arthritis**	↑	Doğruel D, et al. [31]
**Systemic sclerosis**	↑	Guillen-Del Castillo A, et al. [32]
**Sarcoidosis**	↑	Choi J, et al. [33]
**Intestinal bowel disease (IBD)**	↑	Furlano RI, et al. [34]

**Table 2:** Factors that may perturb FeNO measurements.

**FRACTION OF EXHALED NITRIC OXIDE (FENO) AND INTERFERRAL FACTORS**		
**INCREASED FeNO**	**DECREASED FeNO**	**STUDIES**
**Drug intake:**	**Drug intake:**	Sumino H, et al [35], Holden WE, et al [36], Runer T, et al [37], Sippel JM, et al [38], Yates DH, et al [39]
(i) ACE inhibitors [35];	(i) Oxymetazoline [38];	
(ii) Papaverin [36];	(ii) NOS inhibitors [39];	
(iii) Sodium nitroprusside [37].		
**Food and Beverages:**	**Food and Beverages:**	McKnight GM, et al [40], Tenero L, et al [41]
(i) Nitrite/Nitrate-enriched food [40];	(i) Curcumin and Resveratrol [41]	
(ii) Arginine ingestion [40].		
**Environmental factors:**	**Environmental factors:**	Binding N, et al [42], Franklin P, et al. [43], Nightingale JA, et al [44]
(i) NO in air [42];	(i) Calibration of FeNO analyser [42];	
(ii) Calibration of FeNO analyser [43];		
(iii) Occupational exposure to ozone, chlorine dioxide, formaldehyde [44];		
**Physiological and pathological conditions and tests:**	**Physiological and pathological conditions and tests:**	Kharitonov SA, et al [45], Liu PF, et al [46], Chen FJ, et al [47], Uppalapati A, et al [48], Song WJ, et al [49], Xu X, et al [50], Yates DH, et al [51], Dimov PK, et al [52], Deykin A, et al [53], Piacentini A, et al [54], Pendergast DR, et al [55], Phillips CR, et al [56], Kharitonov SA, et al [57], Tang S, et al [58], Murri V, et al [59]
(i) Airway Infections [45];	(i) Tabagism [50];	
(ii) Lung neoplasia [46];	(ii) Alcohol ingestion [51];	
(iii) COPD [47];	(iii) Chronic hypoxia [52];	
(iv) Asthma [47];	(iv) Spirometry [53];	
(v) Systemic inflammatory conditions [48];	(v) Sputum induction [54];	
(vi) Chronic cough [49].	(vi) Hypothermia [55];	
	(vii) Hyperventilation [53];	
	(viii) Physical exercise [56];	
	(ix) Menstrual cycle [57];	
	(x) Childhood [58];	
	(xi) Female gender [59].	
Sickle anemia [60], circadian rhythm [61], and pregnancy [62] are actually under consideration		Cohen RT, et al [60], Saito J, et al [61], Nittner-Marszalska M, et al [62]

## Data Availability

We searched Cochrane Central Register of Controlled Trials (CENTRAL), PubMed, MEDLINE, Embase, and Web of Science.

## Abbreviations

ATS: American Thoracic Society
BMI: Body mass index
CANO: Alveolar concentration of NO
CD: Crohn's disease
CHF: Congestive heart failure
COPD: Chronic obstructive pulmonary disease
ERS: European Respiratory Society
FeNO: Fraction of exhaled nitric oxide
FVC: Force vital capacity
FEV1: Force expiratory volume in the 1st second
FEF: Forced expiratory flow
HL: Hodgkin's Lymphoma
HPS: Hepato-pulmonary syndrome
IBD: Inflammatory bowel disease
IL: Interleukin
NF*κ*B: Nuclear factor kappa-light-chain-enhancer of activated B cells
NO: Nitric oxide
NOS: Inducible form of nitric oxide synthase: (i) e: endothelial, (ii) i: inducible, (iii) mt: mithocondrial, and (iv) n: neuronal
OSAS: Obstructive sleep apnea syndrome
PASI: Psoriasis Area Severity Index
PH: Pulmonary hypertension
PS: Psoriasis
SSc: Systemic sclerosis
TNF: Tumour necrosis factor-*α*.
